# Cell type–specific paradox of autophagy and senescence in MASH: implications for precision hepatology

**DOI:** 10.3389/fcell.2026.1837383

**Published:** 2026-05-13

**Authors:** Md Entaz Bahar, Deok Ryong Kim

**Affiliations:** Department of Biochemistry and Convergence Medical Sciences and Institute of Medical Science, Gyeongsang National University College of Medicine, Jinju, Republic of Korea

**Keywords:** autophagy, hepatic stellate cells, hepatocytes, MASH, senescence

## The spatial challenge in MASH

Metabolic dysfunction–associated steatohepatitis (MASH) has emerged as a major global health burden characterized by progressive lipid accumulation, chronic inflammation, and fibrotic remodeling of the liver. Despite rapid advances in metabolic therapeutics, effective strategies to halt disease progression remain limited, partly due to incomplete understanding of the complex cellular stress programs that govern disease development. Among these mechanisms, the relationship between autophagy and cellular senescence has attracted increasing attention. In many biological contexts these pathways are described as a “double-edged sword,” reflecting their context-dependent roles in cellular protection and pathology (Bahar et al., 2026; Gewirtz, 2013; Gewirtz, 2014). However, in the heterogeneous cellular environment of the liver, this apparent paradox may arise largely from cell-type–specific differences in how these pathways function within the hepatic microenvironment.

**FIGURE 1:**
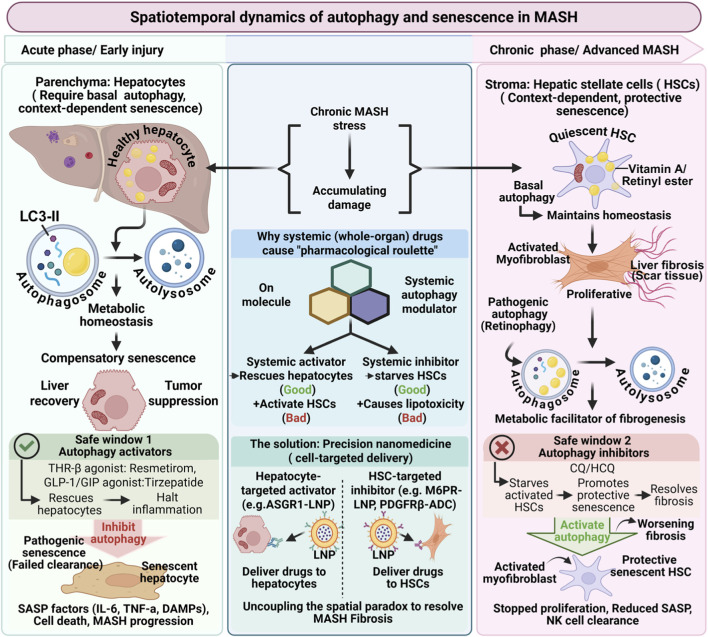
Spatiotemporal dynamics of autophagy and cellular senescence in MASH progression. The progression of metabolic dysfunction–associated steatohepatitis (MASH) is influenced by distinct spatiotemporal dynamics, characterized by sometimes opposing and time-dependent responses to autophagy and cellular senescence among different hepatic cell populations. (Left panel). Hepatocyte compartment. In hepatocytes, canonical degradative autophagy, particularly lipophagy and mitophagy, functions as a critical metabolic quality-control system. By facilitating the degradation of lipid droplets and damaged mitochondria, autophagy limits lipotoxic stress and reactive oxygen species accumulation. During early or acute injury, stress-induced hepatocyte senescence can act as a compensatory, tumor-suppressive safeguard to limit the proliferation of severely damaged cells. However, persistent impairment of these quality-control pathways during chronic disease may contribute to metabolic stress, hepatocyte dysfunction, and the development of senescence-associated inflammatory signaling. Therapeutic strategies that restore metabolic homeostasis in hepatocytes, including metabolic modulators such as THR-β agonists or incretin-based therapies, may partially act through mechanisms that support cellular quality control pathways. (Right panel) Hepatic stellate cell compartment. In contrast, while quiescent hepatic stellate cells (HSCs) maintain a basal level of autophagy essential for cellular homeostasis, stress-induced upregulation of autophagy contributes to the metabolic reprogramming required for fibrogenic activation. During stellate cell activation, autophagic degradation of retinyl ester lipid droplets (often termed retinophagy) provides metabolic substrates that support extracellular matrix production and are strongly associated with fibrogenesis. In this cellular context, entry into cellular senescence may limit fibrogenic activity and facilitate immune-mediated clearance of activated stellate cells. (Bottom) Conceptual implication: Spatial therapeutic challenge. Because hepatocytes and stellate cells respond differently to autophagy and senescence modulation over the course of disease progression, systemic pharmacological interventions targeting these pathways may produce mixed or unintended outcomes. This conceptual framework highlights the potential importance of cell-type–specific therapeutic strategies, including targeted drug delivery systems (e.g., ligand-directed nanoparticles or antibody–drug conjugates) designed to selectively modulate these pathways in specific hepatic cell populations. This figure was created with BioRender.com. (Abbreviations: MASH, Metabolic dysfunction-associated steatohepatitis; SASP, Senescence-Associated Secretory Phenotype; HSC, Hepatic Stellate Cell; LNP, Lipid Nanoparticle; ADC, Antibody-Drug Conjugate)*.*

Current therapeutic approaches often treat the liver as a homogeneous organ and attempt to modulate autophagy or senescence at the whole-organ level. In practice, this strategy faces conceptual challenges because different liver cell populations may respond to the same intervention in fundamentally different ways. We propose that the apparent paradox of autophagy and senescence in MASH is driven primarily by spatial heterogeneity among hepatic cell types, although disease stage and temporal progression also contribute to these dynamics. Recognizing this spatial complexity may help explain why systemic modulation of these pathways has shown limited translational success and highlights the need for cell-targeted therapeutic strategies (Figure 1).

## Hepatocytes: autophagy as a protective metabolic safeguard

In hepatocytes, autophagy plays a critical cytoprotective role in maintaining metabolic homeostasis. Specialized forms of autophagy such as lipophagy and mitophagy help remove excess lipid droplets and damaged mitochondria, thereby limiting lipotoxic intermediates and reactive oxygen species (ROS) accumulation (Singh et al., 2009).

During the early stages of metabolic dysfunction-associated steatotic liver disease (MASLD), chronic nutrient excess and metabolic stress can overwhelm this protective autophagic capacity. Impaired lipophagy leads to lipid droplet accumulation, mitochondrial dysfunction, and oxidative stress. Furthermore, persistent impairment of these autophagic quality-control pathways during chronic disease has been directly linked to the development of hepatocyte senescence and metabolic dysfunction (Ma et al., 2022). Persistent cellular stress may eventually trigger hepatocyte senescence through pathways involving p53/p21 or p16-mediated cell cycle arrest (Bahar et al., 2026). In the acute phase of liver injury, this senescence response serves a highly beneficial, tumor-suppressive function by preventing the proliferation of severely damaged hepatocytes and limiting malignant transformation to hepatocellular carcinoma (HCC) (Patel et al., 2020; Wang et al., 2018). However, as the disease progresses to chronic MASH, this dynamic flips. The prolonged accumulation and failed clearance of these cells becomes deeply detrimental, and extensive hepatocyte senescence in end-stage disease serves as a marker for progression to hepatocellular carcinoma (Brunt et al., 2007). Senescent hepatocytes can adopt a pro-inflammatory senescence-associated secretory phenotype (SASP), which promotes immune cell recruitment and amplifies hepatic inflammation (Ogrodnik et al., 2017).

In this context, preservation or restoration of autophagic flux in hepatocytes may represent an important protective mechanism that delays disease progression. Emerging metabolic therapies illustrate this concept. For example, the recently approved thyroid hormone receptor-β agonist Resmetirom improves hepatic lipid metabolism and mitochondrial function, which may indirectly support autophagy-dependent metabolic homeostasis (Harrison et al., 2024; Hong and Choi, 2024). Similarly, incretin hormone-based therapies such as GLP-1 receptor agonists and dual GLP-1/GIP agonists improve insulin sensitivity and systemic metabolic regulation, which may help reduce endoplasmic reticulum stress and metabolic burden in hepatocytes (Loomba et al., 2024; Singh et al., 2024). While the precise mechanistic contribution of autophagy to these therapeutic effects remains an active area of investigation, maintaining hepatocyte quality-control pathways likely contributes to improved metabolic resilience.

## Hepatic stellate cells: context-dependent autophagy and fibrogenic activation

A contrasting, highly context-dependent role of autophagy emerges in hepatic stellate cells (HSCs), the principal drivers of liver fibrosis. In healthy liver tissue, quiescent HSCs maintain a basal level of autophagic flux that is essential for cellular homeostasis, survival, and the regulation of their quiescent state (Raza et al., 2023). These cells safely store vitamin A within retinyl ester lipid droplets. However, upon chronic liver injury or inflammatory signaling, the function of autophagy dynamically shifts. These cells undergo activation and transdifferentiate into proliferative myofibroblasts that synthesize extracellular matrix components and contribute to fibrotic scar formation (Hernández-Gea et al., 2012). This activation process requires substantial metabolic reprogramming. Autophagy, specifically degradation of retinyl ester droplets, sometimes referred to as retinophagy, provides lipid-derived substrates that support the energetic demands of stellate cell activation. Experimental studies have demonstrated that this specific upregulation of autophagic flux contributes to this metabolic transition and is strongly associated with fibrogenesis. Such a dual role of autophagy, maintaining homeostasis in quiescent states while facilitating activation and metabolic reprogramming under stress, is a recognized hallmark of mesenchymal-derived cell populations (Rastaldo et al., 2020).

Interestingly, the functional role of cellular senescence in HSCs differs from that in hepatocytes. Whereas hepatocyte senescence can contribute to inflammatory signaling and disease progression, senescence of activated stellate cells can limit fibrogenic activity. Senescent HSCs reduce collagen synthesis and may become more susceptible to immune-mediated clearance, including removal by natural killer (NK) cells, thereby facilitating fibrosis resolution (Krizhanovsky et al., 2008). Thus, within the hepatic microenvironment, autophagy and senescence can exert opposing functional consequences depending on the cellular context.

## Challenges of systemic modulation of autophagy and senescence

These contrasting cellular responses help explain why systemic pharmacological modulation of autophagy or senescence may produce mixed or unpredictable outcomes in MASH (Bahar et al., 2026). For example, systemic autophagy activation intended to improve hepatocyte lipid metabolism could theoretically enhance autophagic activity in stellate cells and inadvertently support fibrogenic activation. Conversely, broad inhibition of autophagy aimed at suppressing stellate cell activation might impair hepatocyte lipophagy and exacerbate metabolic stress. Similarly, indiscriminate elimination of senescent cells using systemic senolytic agents may remove inflammatory senescent hepatocytes but could also eliminate senescent stellate cells that contribute to fibrosis resolution. These considerations highlight a fundamental limitation of whole-organ pharmacology when targeting pathways that have cell-type-specific roles.

## Toward precision hepatology: cell-targeted therapeutic strategies

Advances in targeted drug delivery offer potential strategies to address this spatial complexity. Emerging approaches such as ligand-directed nanoparticles, antibody–drug conjugates (ADCs), and engineered extracellular vesicles may enable selective delivery of therapeutics to specific hepatic cell populations (Chen et al., 2019). For example, the hepatocyte-specific receptor ASGR1 (asialoglycoprotein receptor 1) has been widely explored as a targeting platform for hepatocyte-directed delivery systems. Such strategies could allow selective delivery of metabolic or autophagy-modulating agents to hepatocytes while minimizing effects on other liver cell populations.

Conversely, activated stellate cells express distinct surface markers such as PDGFRβ or the mannose-6-phosphate receptor, which have been investigated as potential entry points for targeted anti-fibrotic therapies. Selective modulation of stellate cell metabolism or activation state through these pathways may help limit fibrogenesis while preserving beneficial processes in hepatocytes. Although these approaches remain under active development, they illustrate how precision delivery technologies may help uncouple the conflicting biological responses that arise from systemic interventions.

## Beyond hepatocytes and stellate cells: complexity of the hepatic niche

While the hepatocyte–stellate cell axis provides a useful conceptual framework, the hepatic microenvironment includes additional cell populations that contribute to disease progression. Kupffer cells, infiltrating macrophages, liver sinusoidal endothelial cells, cholangiocytes, and immune cells participate in inflammatory signaling, metabolic regulation, and tissue remodeling during MASH. Autophagy and senescence likely influence many of these cell populations as well, and their interactions may further shape disease outcomes. Therefore, a comprehensive understanding of the autophagy–senescence axis in liver disease will require integrated analysis of multiple cell types and their communication within the hepatic niche (Figure 1).

## Conclusion

The relationship between autophagy and cellular senescence in MASH illustrates how biological pathways can produce distinct outcomes depending on cellular context. In hepatocytes, autophagy helps preserve metabolic homeostasis and limit stress-induced senescence, whereas in hepatic stellate cells autophagy supports fibrogenic activation while senescence may contribute to fibrosis resolution. These contrasting roles highlight the limitations of systemic therapeutic strategies targeting these pathways. Future progress in MASH treatment may depend on approaches that account for the spatial heterogeneity of the liver and selectively modulate pathways within specific cell populations. Advances in targeted delivery technologies and precision pharmacology provide promising opportunities to address this challenge. By integrating cell-type-specific biology with emerging drug delivery platforms, it may become possible to harness the beneficial aspects of autophagy regulation and senescence modulation while minimizing unintended effects within the complex hepatic microenvironment.

## References

[B1] Bahar M. E. Hwang J. S. Lai T. H. Akter K. M. Maulidi R. F. Kim D. R. (2026). The autophagy-senescence axis as a threshold model of aging and therapeutic targeting. Redox Biol. 91, 104079. 10.1016/j.redox.2026.104079 41690118 PMC12925196

[B2] Brunt E. M. Walsh S. N. Hayashi P. H. LaBundy J. Di Bisceglie A. M. (2007). Hepatocyte senescence in end-stage chronic liver disease: a study of cyclin-dependent kinase inhibitor p21 in liver biopsies as a marker for progression to hepatocellular carcinoma. Liver Int. 27 (5), 662–671. 10.1111/j.1478-3231.2007.01470.x 17498252

[B3] Chen Z. J. Jain A. Liu H. Zhao Z. Cheng K. (2019). Targeted drug delivery to hepatic stellate cells for the treatment of liver fibrosis. J. Pharmacol. Exp. Ther. 370 (3), 695–702. 10.1124/jpet.118.256156 30886124 PMC6806344

[B4] Gewirtz D. A. (2013). Autophagy and senescence A partnership in search of definition. Autophagy 9 (5), 808–812. 10.4161/auto.23922 23422284 PMC3669198

[B5] Gewirtz D. A. (2014). Autophagy and senescence in cancer therapy. J. Cell. Physiology 229 (1), 6–9. 10.1002/jcp.24420 23794221

[B6] Harrison S. A. Bedossa P. Guy C. D. Schattenberg J. M. Loomba R. Taub R. (2024). A phase 3, randomized, controlled trial of resmetirom in NASH with liver fibrosis. N. Engl. J. Med. 390 (6), 497–509. 10.1056/NEJMoa2309000 38324483

[B7] Hernández-Gea V. Ghiassi-Nejad Z. Rozenfeld R. Gordon R. Fiel M. I. Yue Z. Y. (2012). Autophagy releases lipid that promotes fibrogenesis by activated hepatic stellate cells in mice and in human tissues. Gastroenterology 142 (4), 938–946. 10.1053/j.gastro.2011.12.044 22240484 PMC3439519

[B8] Hong H. Choi J. (2024). A phase 3, randomized, controlled trial of resmetirom in NASH with liver fibrosi. Korean J. Gastroenterology 83 (6), 253–255. 10.4166/kjg.2024.048

[B9] Krizhanovsky V. Yon M. Dickins R. A. Hearn S. Simon J. Miething C. (2008). Senescence of activated stellate cells limits liver fibrosis (vol 134, pg 657, 2008). Cell 135 (1), 190. 10.1016/j.cell.2008.09.015 PMC3073300 18724938

[B10] Loomba R. Hartman M. Lawitz E. Vuppalanchi R. Boursier J. Bugianesi E. (2024). Tirzepatide for the treatment of metabolic dysfunction-associated steatohepatitis with liver fibrosis: results of the SYNERGY-NASH phase 2 trial. J. Hepatology 80, S7–S8. 10.1016/s0168-8278(24)00436-7

[B11] Ma X. W. Williams S. N. Ding W. X. (2022). Linking of senescence to autophagy deficiency in chronic liver disease. Cell. Mol. Gastroenterology Hepatology 14 (2), 405–406. 10.1016/j.jcmgh.2022.04.010 35605640 PMC9304967

[B12] Ogrodnik M. Miwa S. Tchkonia T. Tiniakos D. Wilson C. L. Lahat A. (2017). Cellular senescence drives age-dependent hepatic steatosis. Nat. Commun. 8, 15691. 10.1038/ncomms15691 28608850 PMC5474745

[B13] Patel N. H. Sohal S. S. Manjili M. H. Harrell J. C. Gewirtz D. A. (2020). The roles of autophagy and senescence in the tumor cell response to radiation. Radiat. Res. 194 (2), 103–115. 10.1667/Rade-20-00009 32845995 PMC7482104

[B14] Rastaldo R. Vitale E. Giachino C. (2020). Dual role of autophagy in regulation of mesenchymal stem cell senescence. Front. Cell Dev. Biol. 8, 276. 10.3389/fcell.2020.00276 32391362 PMC7193103

[B15] Raza S. Rajak S. Singh R. Zhou J. Sinha R. A. Goel A. (2023). Cell-type specific role of autophagy in the liver and its implications in non-alcoholic fatty liver disease. World J. Hepatology 15 (12), 1272–1283. 10.4254/wjh.v15.i12.1272 38192406 PMC7615497

[B16] Singh R. Kaushik S. Wang Y. J. Xiang Y. Q. Novak I. Komatsu M. (2009). Autophagy regulates lipid metabolism. Nature 458 (7242), 1131–U1164. 10.1038/nature07976 19339967 PMC2676208

[B17] Singh A. Sohal A. Batta A. (2024). GLP-1, GIP/GLP-1, and GCGR/GLP-1 receptor agonists: novel therapeutic agents for metabolic dysfunction-associated steatohepatitis. World J. Gastroenterology 30 (48), 5205–5211. 10.3748/wjg.v30.i48.5205 39735270 PMC11612699

[B18] Wang C. Chen W. J. Wu Y. F. You P. Zheng S. Y. Liu C. C. (2018). The extent of liver injury determines hepatocyte fate toward senescence or cancer. Cell Death & Dis. 9, 575. 10.1038/s41419-018-0622-x 29760381 PMC5951829

